# Which parent, which schema mode? Examining the role of parental gender in adverse childhood experiences

**DOI:** 10.1186/s40359-025-03825-3

**Published:** 2025-12-30

**Authors:** Soda Nematzadeh, Adel Fatemi, Elham Gheysvandi, Farideh Nargesi

**Affiliations:** 1 Department of Psychology, Sa.C., Islamic Azad University, Sanandaj, Iran; 2 Department of Statistics, Sa.C., Islamic Azad University, Sanandaj, Iran; 3 Department of Public Health, Sa.C., Islamic Azad University, Sanandaj, Iran; 4 Department of Clinical Psychology, Sa.C., Islamic Azad University, Sanandaj, Iran

**Keywords:** Early maladaptive schemas, Schema modes, Adverse childhood experiences, Parental gender, Emotional relationships

## Abstract

**Background:**

Schema modes represent momentary cognitive, emotional, behavioral, and neurobiological states that are activated in response to environmental experiences. They develop through the interaction between a child’s temperament and early adverse family conditions. This study examined the mediating role of parental gender in the relationship between adverse childhood experiences (ACEs) and schema modes.

**Methods:**

A quantitative cross-sectional design was employed with a sample of 100 adults receiving psychotherapy in Sanandaj, Iran, during autumn 2023. Participants were selected through random sampling. Data were collected using the Adverse Childhood Experiences Questionnaire, the Schema Mode Inventory, and the Family Emotional Climate Scale. Partial Least Squares Structural Equation Modeling (PLS-SEM) with bootstrapping procedures was used for data analysis.

**Results:**

Significant pathways were found from ACEs to both mother–child and father–child emotional relationships (*p* < .001). The Mother–child emotional relationship showed a significant association with schema modes (*p* < .001), whereas the father–child relationship did not (*p* > .05). In the male subgroup, none of the paths to schema modes reached statistical significance (all *p* > .05). These findings reflect associations rather than causal effects.

**Conclusion:**

Findings suggest that the emotional quality of the mother–child emotional relationships were more strongly associated with schema modes than father–child relationships at the sample level. No significant paths to schema modes were observed in the male subgroup, indicating that conclusions about gender-specific influences should remain cautious and exploratory. Further research using formal multi-group analyses is required to clarify potential gender-related differences.

## Background

Schema therapy integrates theoretical and practical principles from multiple psychological traditions, including cognitive-behavioral, psychodynamic, experiential, and Gestalt approaches. Due to its integrative framework and growing empirical support, schema therapy has become increasingly utilized in clinical practice, particularly for individuals with complex personality difficulties and chronic emotional problems rooted in early adverse experiences [1].

Schemas represent enduring cognitive–emotional structures that guide how individuals perceive themselves, others, and the world. These schemas are shaped through early life experiences and can be either adaptive or maladaptive. Maladaptive schemas often originate in childhood and persist into adulthood, influencing emotional regulation and interpersonal functioning even when they are no longer relevant to current circumstances [2–4].

As conceptualized by Young, schemas are not merely thoughts but broad cognitive–emotional templates formed through the interaction between life experiences, affective responses, and physiological reactions [5]. When these templates become rigid, they may give rise to dysfunctional behaviors and emotional distress. Schema therapy addresses such maladaptive beliefs and emotional patterns by helping individuals identify and modify early negative schemas through cognitive restructuring, emotional processing, and behavioral change techniques [6].

To provide a more dynamic understanding of personality functioning, the concept of *schema modes* was introduced as an extension of schema theory [7]. Schema modes refer to transient emotional, cognitive, and behavioral states that become activated in response to environmental triggers. These modes represent momentary configurations of multiple schemas and coping styles that interact in specific situations. Dysfunction occurs when certain maladaptive modes become dominant, leading to distress and self-defeating behavior patterns [8–10].

Core cognitive assumptions about the self and others are formed through the interaction between a child’s innate temperament and early caregiving experiences. Harsh or neglectful parenting practices can disrupt this developmental process and foster the formation of maladaptive schemas [11–13]. The *Diagnostic and Statistical Manual of Mental Disorders (DSM)* defines adverse childhood experiences (ACEs) as traumatic events or chronic stressors occurring before the age of 18, encompassing three primary domains: childhood abuse (emotional, physical, and sexual), neglect (emotional and physical), and family dysfunction (e.g., domestic violence, substance abuse, parental separation, mental illness, or incarceration of a family member) [9, 14].

A growing body of evidence indicates that ACEs play a crucial role in the emergence of early maladaptive schemas (EMSs) and schema modes throughout the lifespan. Experiences such as emotional neglect, emotional and physical abuse, and overprotective parenting have been consistently linked to maladaptive schema development in adolescence and adulthood [15–17]. A 2020 systematic review and meta-analysis confirmed that ACEs are robust predictors of EMSs in adulthood, particularly within the domains of disconnection, impaired autonomy, and over-vigilance [15]. Similarly, a 2022 review highlighted that overprotective parenting—even in the absence of overt abuse—can contribute to maladaptive schema formation across developmental stages [16].

Building on this evidence, recent schema therapy research emphasizes the critical role of early family dynamics in shaping schemas and schema modes. However, despite extensive attention to parenting behaviors, relatively few studies have investigated how parental gender specifically influences schema mode development [18, 19]. A 2022 expert consensus identified this topic as a high-priority area for future schema therapy research [13].

Moreover, emerging literature suggests that parents’ own maladaptive schemas may be transmitted intergenerationally, influencing their children’s emotional adjustment and cognitive representations [20, 21]. This intergenerational process underscores the central role of parenting behaviors and family emotional environments in shaping the child’s cognitive–emotional development. In Iranian families, caregiving responsibilities are often gendered: mothers typically provide primary emotional care, while fathers are more engaged in financial and structural roles. These culturally embedded patterns may influence how maternal and paternal relationships contribute differently to schema mode formation.

Given these theoretical and cultural considerations, the current study aims to examine whether parental gender (mother or father**)** moderates the relationship between adverse childhood experiences and schema modes in adulthood. Specifically, this study explores how distinct maternal and paternal influences shape the emergence of maladaptive schema modes, offering culturally contextualized insights into gendered parent–child dynamics within the schema therapy framework [22].

### Implications of the study

This research examines the relationship between negative childhood experiences and schema modes, with a particular focus on parental gender differences in their impact on schema development. The findings can guide psychologists and therapists in schema therapy to pay closer attention to parental gender effects and tailor therapeutic approaches accordingly. Addressing these research priorities provides critical insights that can advance schema therapy as a transdiagnostic approach for treating chronic mental health disorders.Understanding these gender-specific influences requires considering both caregiving roles and cultural expectations, which are further explored below.

Early parental influences play a critical role in the formation of schema modes, with maternal relationships often exerting a stronger impact than paternal ones. This pattern reflects both caregiving roles and cultural expectations, shaping how children internalize emotional experiences. Highlighting these gendered differences earlier in the text situates the study within a clear theoretical framework, emphasizing how distinct parent-child dynamics contribute to the development and activation of early maladaptive schemas, and providing context for interpreting the observed findings.

## Methods

### Study design

**Fig. 1:**
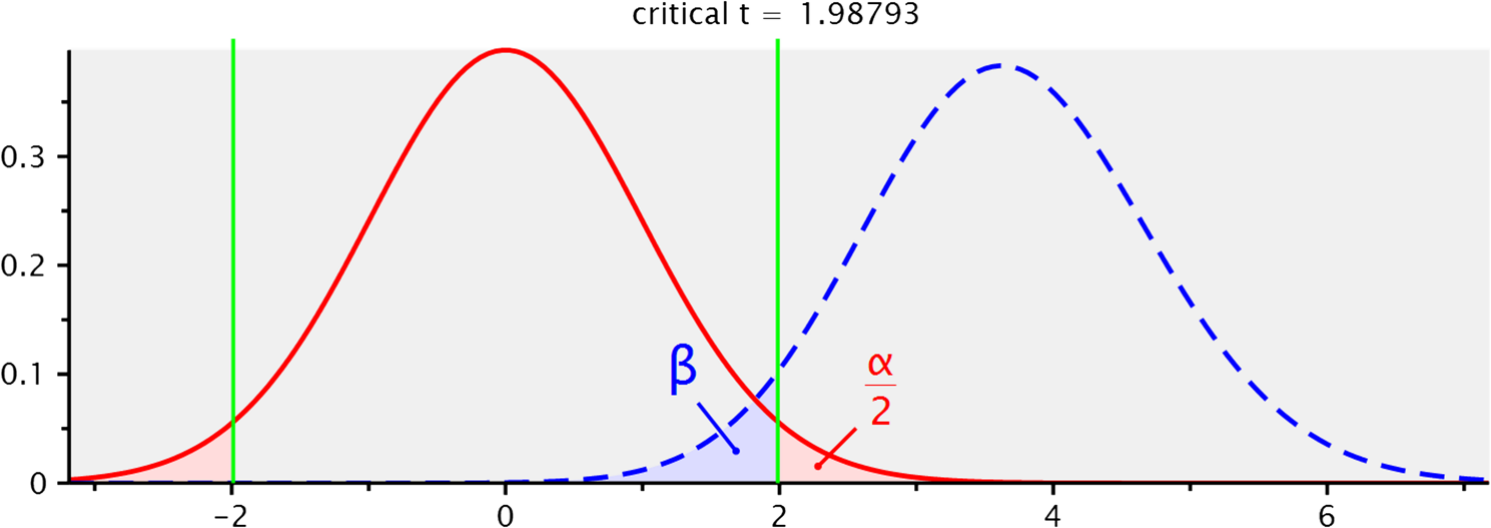
Results of the a priori power analysis using G*Power. Displays the G*Power output for the a priori analysis, confirming a required sample size of N=89 to achieve 95% statistical power (1-beta =0.95) for detecting a medium effect size (f2 =0.15) with two predictors and an alpha level of 0.05. t tests - Linear multiple regression: Fixed model, single regression coefficient. Analysis: A priori: Compute required sample size. Input: Tail(s) = Two. Effect size f² = 0/15. α err prob = 0/05. Power (1-β err prob) = 0/95. Number of predictors = 2. Output:Noncentrality parameter δ = 3/6537652. Critical t = 1/9879342. Df = 86. Total sample size = 89. Actual power = 0/9508043

This quantitative study utilized a cross-sectional design and was conducted in the fall of 2023 in the city of Sanandaj, Iran. The cross-sectional nature of the study, while efficient for surveying current relationships, is a limitation as it prevents drawing causal inferences between the variables. The study population included all individuals who sought psychological therapy services at accessible psychotherapy clinics in Sanandaj. Using G-Power software, the minimum sample size required for the study was calculated to be 89 participants (Fig. 1). To ensure robustness, 100 individuals were randomly selected from the clinic attendees as the final sample.

The inclusion criteria for participants were seeking psychological therapy using a random number generator services at Sanandaj psychotherapy clinics, being over 19 years old, and providing informed consent to participate in the study. The exclusion criteria were refusal to participate in the study, and being under 19 years old.

The study employed three standardized questionnaires to collect data: the Schema Mode Inventory, the Adverse Childhood Experiences (ACEs) questionnaire, and the Family Emotional Climate Scale.

ACEs Questionnaire developed by Felitti et al. in 1998, this 10-item questionnaire assesses the relationship between adverse childhood experiences and health outcomes in adulthood for individuals aged 19 and older. It has a binary scoring system (“Yes” = 1 point, “No” = 0 points). The total score reflects the number of adverse experiences but not their severity. Five items address child maltreatment, and five concern parental or familial problems. Higher scores indicate a greater number of adverse experiences [23].a study examined the psychometric properties of the 10-item version of the Adverse Childhood Experiences questionnaire (ACE-10) among Hungarian adolescents. The findings indicated that the tool demonstrated acceptable internal consistency (α = 0.64) and showed good concurrent validity with the Strengths and Difficulties Questionnaire (SDQ) and the HBSC Symptom Checklist [24]. In Iran, Badami et al. (2020) assessed its reliability, reporting Cronbach’s alpha coefficients of 0.758 and 0.702 [25].

The short-form Schema Mode Inventory, developed by Lobbestael et al. in 2010, contains 124 items and assesses 14 schema modes: Maladaptive modes (e.g., Vulnerable Child, Angry Child, Impulsive Child, Demanding Parent, Punitive Parent), Adaptive modes (e.g., Happy Child, Healthy Adult), Coping modes (e.g., Compliant Surrenderer, Detached Protector, Overcompensator). the Persian short version of the Schema Mode Inventory demonstrated excellent internal consistency, with a Cronbach’s alpha of 0.94 [26].

Family Emotional Climate Scale questionnaire, This 16-item questionnaire developed by Hilburn in 1964, measures family emotional climate (e.g., parent-child relationships, emotional security, and trust). Items are rated on a five-point Likert scale (e.g., “I felt secure in my relationship with my father”). It evaluates eight dimensions, including affection, encouragement, shared experiences, and security.Nahidi assessed its content, criterion, and face validity, finding an overall reliability efficient above 0.7 using Cronbach’s alpha.In related studies, predictive validity showed significant correlations with parents’ and children’s psychological problems (*r* =.28, *p* <.01; *r* =.41, *p* <.001) [27, 28].

### Methodological justification

The decision to employ the Partial Least Squares Structural Equation Modeling)PLS-SEM) approach was a strategic methodological choice, driven by a rigorous evaluation of both our research objectives and the empirical characteristics of the data.

This method was selected over covariance-based alternative) CB-SEM) because its core philosophy aligns perfectly with the study’s central goal: the prediction and maximal explanation of variance in the endogenous constructs Schema-Related Modes and Family Emotional Climate. As PLS-SEM is inherently a causal-predictive technique, it represents the most appropriate tool for achieving our theoretical objectives [29].

Furthermore, the nature of our collected data strongly validated this approach. As clearly documented by the findings in Tables 1 and 2, the observed non-normal distribution of several key study variables necessitated a robust analytical solution. PLS-SEM is a non-parametric method that does not impose the restrictive assumption of multivariate normality, which provided a crucial methodological advantage. Finally, while the adequacy of our sample size (*N* = 100) was confirmed via G*Power analysis (Fig. 1), PLS-SEM is recognized as being more resilient and reliable than CB-SEM when analyzing relatively complex structural models with smaller sample sizes, thereby guaranteeing the stability of our structural path estimates.

**Table 1:** Results of normality testing for key latent variables. Summarizes the outcomes of normality tests (e.g., Kolmogorov-Smirnov/Shapiro-Wilk) alongside the mean, standard deviation, and significance level (Sig.) for the three main variables to determine which follow a normal distribution, justifying the use of PLS -SEM

Variable	Mean	Standard Deviation	Sig	Result
Adverse Childhood Experiences	1.51	1.77	0.000	Not Normal
Schema Modes	2.65	0.41	0.012	Not Normal
Father-Child Emotional Climate	2.92	1.08	0.200	Normal
Mother-Child Emotional Climate	3.17	1.06	0.001	Not Normal

**Table 2:** Skewness and Kurtosis Values for Schema Modes subscales. This table presents descriptive statistics for the subscales of the Schema Mode Inventory, specifically showing the skewness and kurtosis values to supplement the Kolmogorov-Smirnov test in assessing the shape and symmetry of the data distribution

Subscale	Mean	Sig	Result
Vulnerable Child	2.64	0.032	Not Normal
Angry Child	2.59	0.001	Not Normal
Enraged Child	1.79	0.003	Not Normal
Impulsive Child	1.64	0.003	Not Normal
Undisciplined Child	5.79	0.200	Normal
Happy Child	1.75	0.001	Not Normal
Submissive	2.71	0.001	Not Normal
Detached Protector	2.63	0.001	Not Normal
Detached Self-Soother	1.41	0.001	Not Normal
Self-Comforter	6.91	0.000	Not Normal
Self-Aggrandizer	2.20	0.001	Not Normal
Bully	2.17	0.004	Not Normal
Punitive Parent	1.95	0.000	Not Normal
Demanding Parent	3.05	0.024	Not Normal
Healthy Adult	4.02	0.200	Normal

**Fig. 2:**
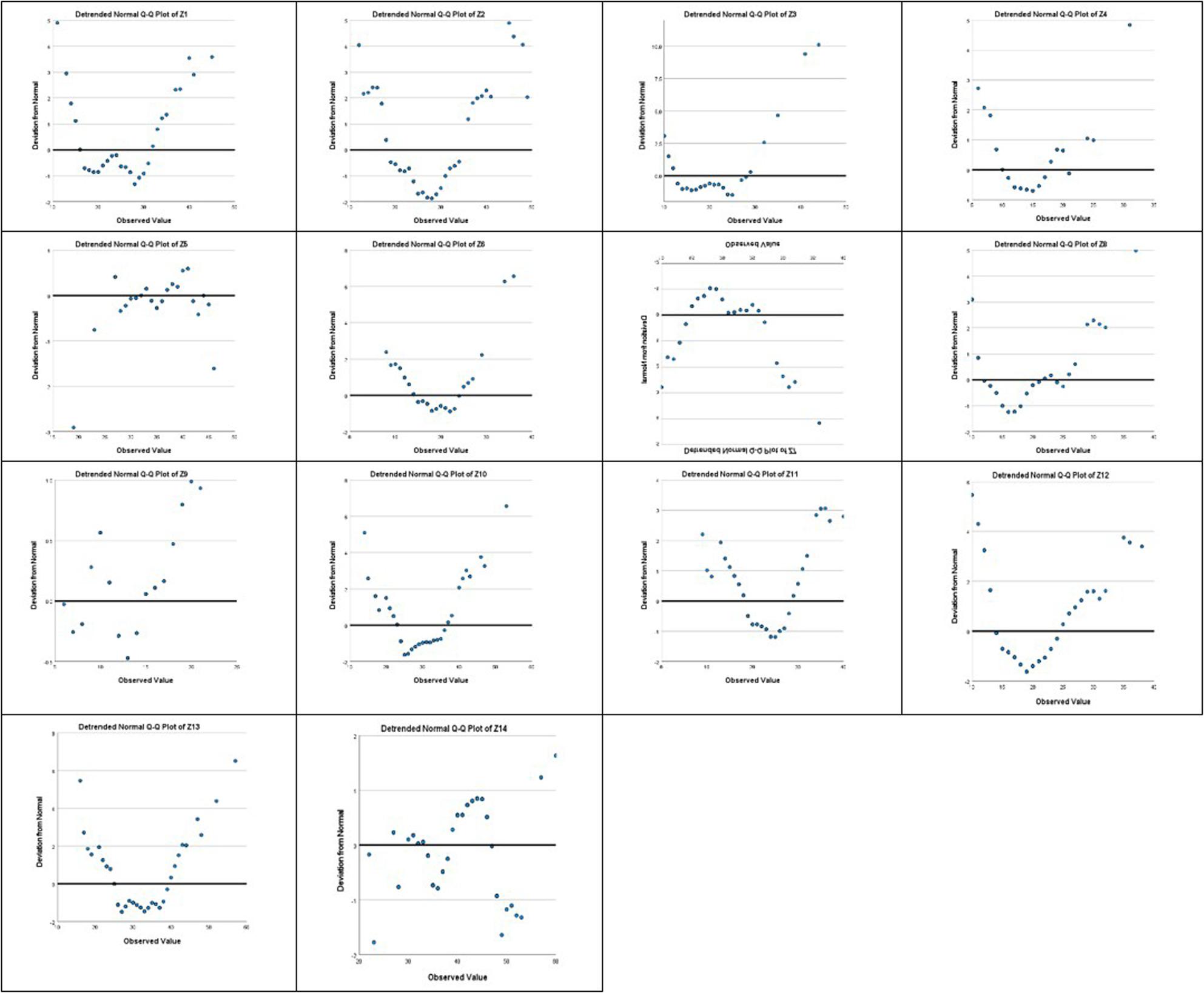
Multiple Q-Q plots for visual normality assessment of Schema Mode subscales. Shows the Q-Q plots used for the visual evaluation of data distribution across various schema mode subscales, where the alignment of data points with the diagonal line suggests proximity to a normal distribution

**Fig. 3:**
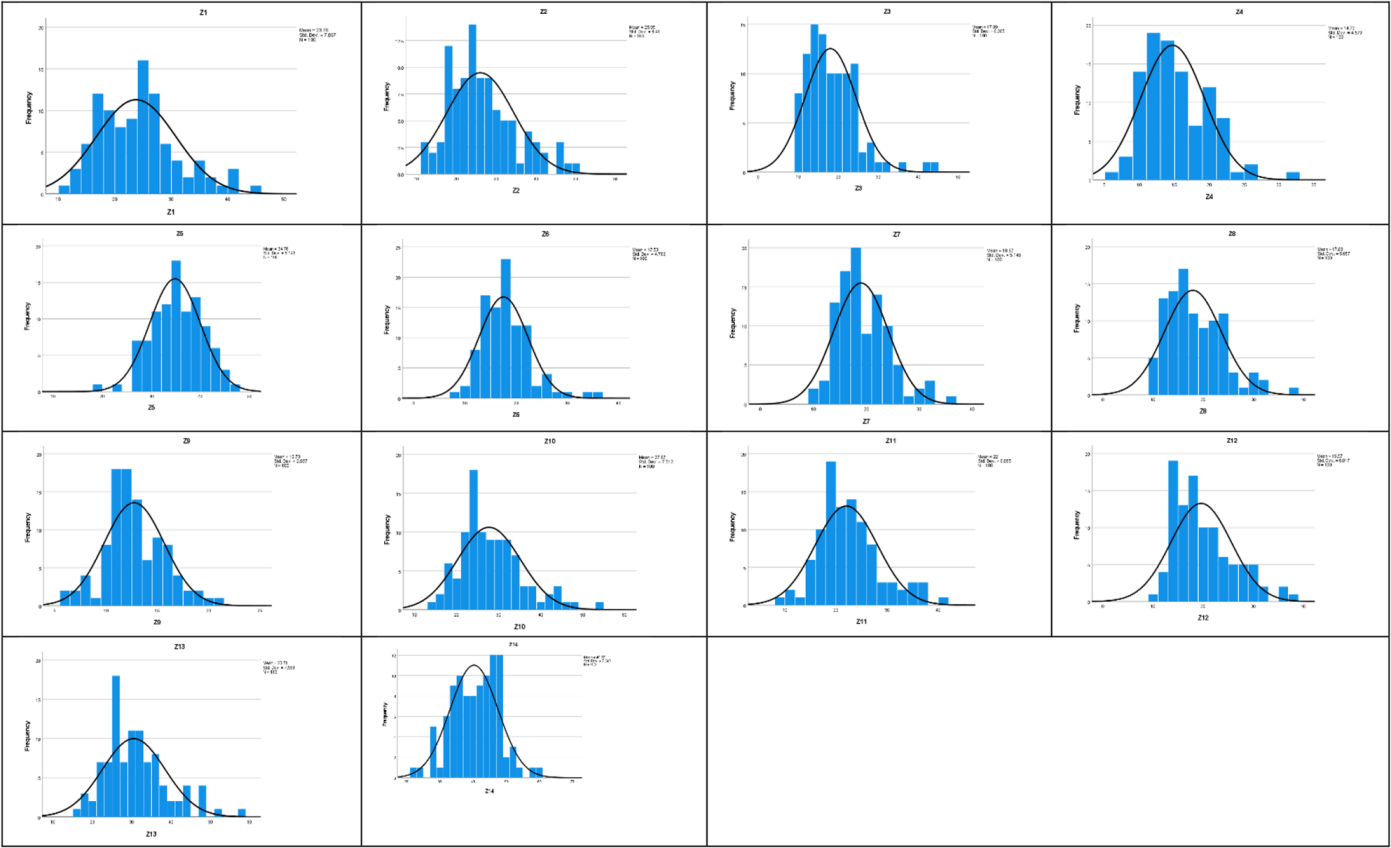
Histograms illustrating the distribution of Schema Modes. Presents the frequency histograms for the total scores of schema modes, providing a visual representation of the empirical data distribution to assess the degree of deviation from a normal distribution, supplementing the statistical normality tests

Normality of the research variables was assessed using multiple approaches. Initially, the Kolmogorov–Smirnov (K–S) test was conducted (as seen in Table 2); however, given its limitations in small and moderate sample sizes, complementary indicators were also considered. Skewness and kurtosis values were examined for all observed variables, with several variables (e.g., Z1 to Z14) showing skewness and/or kurtosis exceeding conventional thresholds (± 1), indicating deviation from normality (as seen in Table 3). Visual inspections of histograms and Q–Q plots were also performed for the primary variables. These graphical assessments confirmed that most variables did not follow a perfectly normal distribution (Figs. 2 and 3). Accordingly, the data were considered non-normal.

**Table 3:** Descriptive statistics (Skewness, and Kurtosis) of Schema Mode subscales.This table presents the shape parameters (Skewness and Kurtosis) for all subscales of the Schema Mode Inventory. These statistics are utilized to formally evaluate the distribution characteristics of each mode, supplementing the Kolmogorov-Smirnov test results and informing the decision to employ the PLS-SEM approach for non-normal data

Schema modes	*N*	Skewness		Kurtosis	
	Statistic	Statistic	Std. Error	Statistic	Std. Error
Z1	100	.678	.241	.275	.478
Z2	100	.792	.241	.261	.478
Z3	100	1.421	.241	3.263	.478
Z4	100	.676	.241	.502	.478
Z5	100	-.147	.241	-.040	.478
Z6	100	1.116	.241	2.514	.478
Z7	100	.810	.241	.667	.478
Z8	100﻿	.828	.241	.491	.478
Z9	100	.293	.241	.323	.478
Z10	100	.848	.241	.835	.478
Z11	100	.711	.241	.602	.478
Z12	100	.930	.241	.374	.478
Z13	100	.930	.241	.761	.478
Z14	100	-.098	.241	-.109	.478
Valid N (listwise)	100				

Despite the observed non-normality, Partial Least Squares Structural Equation Modeling (PLS-SEM) was employed for subsequent analyses. PLS-SEM is robust to deviations from normality and is particularly suitable for complex models with multiple latent constructs and small to medium sample sizes [29]. The variables Adverse Childhood Experiences, Schema Modes, and Mother-Child Emotional Climate exhibited non-normal distributions (Sig < 0.05). Conversely, the Father-Child Emotional Climate variable showed a normal distribution (Sig >0.05) (as seen in Table 1). Similarly, among the Schema Modes subscales, only the Undisciplined Child and Healthy Adult subscales exhibited normal distributions, while all others were non-normally distributed (as seen in Table 2). The prevalence of non-normal distributions across key variables necessitated the use of a non-parametric-friendly technique like PLS-SEM, which is robust to deviations from normality, thus confirming the validity of our methodological choice [29].

The Goodness-of-Fit (GOF) index for the model was calculated as 0.282. Based on established thresholds (0.01, 0.25, and 0.36 for weak, moderate, and strong fit, respectively), this value suggests a moderate fit for the proposed structural model [29].

The research protocol was approved by the Research Ethics Committee of Sanandaj Islamic Azad University of Medical Sciences (approval code: IR.IAU.SAJ.REC.1402.11.1). Researchers visited psychotherapy clinics in Sanandaj, explained the study’s goals, and obtained written informed consent from participants. Participants then completed the questionnaires. To ensure ethical compliance, participants were assured of confidentiality, and results were reported in aggregate form.

## Results

The analytical phase began by summarizing the data through descriptive statistics, computing measures of central tendency (mean, median, mode) and dispersion (variance, standard deviation, and range), alongside frequency distributions to characterize both the sample and the research metrics. Given the study’s primary causal-predictive objective and the specific features of the data, the hypotheses were rigorously tested using Structural Equation Modeling)SEM) via the Partial Least Squares)PLS) approach, implemented in SmartPLS 4.0. This methodological choice was strategic, primarily driven by the observed non-normal distribution of most variables (as confirmed by the Kolmogorov-Smirnov test) and the relatively small sample size *N* = 100). These conditions rendered PLS-SEM significantly more robust and appropriate than covariance-based alternatives like AMOS or LISREL [29].

To ensure the statistical integrity and robustness of the estimates, the bootstrapping procedure—a non-parametric resampling technique—was rigorously implemented. Following the established methodological recommendations of Hair et al. (2017), a total of 5,000 resamples were generated. This crucial step yielded highly reliable standard errors and more accurate confidence intervals for the path coefficients, which was essential given the evidence of non-normal distribution observed in several key study variables [29].

The study sample consisted of 100 participants, with a mean age of M = 33.98 years (M = 33.98, SD = 8.26). Regarding educational background, 15% of the participants held a high school diploma, 30% had an associate degree, 43% a bachelor’s degree, 35% a master’s degree, and 4% a doctoral degree. In terms of gender distribution, 77% were female and 23% male (as seen in Table 4).

**Table 4:** Demographic characteristics of study participants (*N*=100) : presents the distribution of key demographic variables, including the mean and standard deviation of age, and the frequency and percentage distribution of education level and gender (female and male) for the entire sample (*N*=100)

Variable	Sample Size	Mean Age	Age Standard Deviation	
Age	100	33.98	8.26	
Education Level			Frequency	Percentage
High school diploma			4	4%
Associate degree			15	15%
Bachelor's degree			42	42%
Master's degree			35	35%
Doctorate			4	4%
Gender			Frequency	Percentage
Female			77	77%
Male			23	23%

The mean score for Adverse Childhood Experiences (ACE) was relatively low **(**M = 1.51, SD = 1.90**)**, suggesting limited exposure to severe early adversities within the sample. Schema Modes demonstrated moderate-to-high activity levels **(**M = 2.65, SD = 0.81**)** on a five-point Likert scale, indicating moderate engagement with schema-related emotional patterns. The rank orders of the schema modes are presented (Table 5). Family Emotional Climate scores reflected generally positive relationships, with the Mother–Child relationship (M = 3.17, SD = 0.52) slightly higher than the Father–Child relationship (M = 2.92, SD = 0.59), implying stronger perceived maternal emotional bonds (as seen in Table 6). The data analysis was conducted in two primary steps: Measurement Model Evaluation (assessing validity and reliability) and Structural Model Evaluation assessing explanatory power and hypothesis testing).

**Table 5:** Rank orders of schema modes based on mean scores and regression-derived t-values

mode	Mean	Rank (Mean)	Std. Deviation	T-value	Rank (T-valu)
Healthy Adult	40.27	1	7.243	5.33	12
Undisciplined Child	34.76	2	5.143	2.91	13
Demanding Parent	30.51	3	7.996	6.64	9
Self-Aggrandizer	27.67	4	7.512	6.48	10
Angry Child	25.95	5	8.410	23.28	2
Vulnerable Child	23.76	6	7.067	26.53	1
Overcompensator	22.00	7	6.085	9.18	8
Punitive Parent	19.57	8	6.017	20.35	4
Compliant Surrenderer	18.97	9	5.149	9.91	7
Enraged Child	17.99	10	6.365	10.26	6
Detached Protector	17.88	11	5.657	10.49	5
Happy Child	17.53	12	4.766	6.01	11
Impulsive child	14.72	13	4.579	21.61	3
Detached Self-Soother	12.73	14	2.937	2.51	14

The Schema Modes were ranked based on two criteria: the Mean score (M reflecting Perceived intensity, and the *T*-Values derived from the Outer Loadings in the measurement model, which reflect the relative importance and reliability of the mode's indicator, SD =Standard Deviation

**Table 6:** Descriptive statistics (mean and standard deviation) for primary study variables: reports the descriptive statistics, specifically the Mean (M) and Standard Deviation (SD), for the main latent variables: Adverse Childhood Experiences (ACEs), Schema Modes, and the components of the family emotional climate (mother-child relationship and father-child relationship)

Variable	Mean	Standard Deviation
Adverse Childhood Experiences	1.51	1.77
Schema Modes	2.65	0.41
Family Emotional Climate:		
- Father-child relationship	2.92	1.08
- Mother-child relationship	3.17	1.06

The measurement model was examined to ensure that each latent construct was measured reliably and validly by its corresponding indicators.

### Convergent validity and internal consistency

Convergent validity was confirmed through the evaluation of factor loadings, Average Variance Extracted (AVE), and Composite Reliability (CR).

All constructs exhibited satisfactory factor loadings (above 0.40) and significant *t*-values (t > 1.96), thereby supporting convergent validity. For example, items for ACE ranged from 0.305 to 0.789 (all p *<*.05), while Schema Modes (e.g., Abandoned/Abused and Angry Child subconstructs) showed loadings above 0.70. Similarly, Parent–Child Relationship indicators (JA1–JA14) loaded between 0.446 and 0.848 (as seen in Table 7).

**Table 7:** Standardized factor loadings and *t*-statistics for the convergent validity of the measurement mode

Factors/Items	Factor Loading	*t*-Statistic	Significance Level	Result
ACE1 <- Adverse Childhood Experiences	0.569	4.970	0.000	Confirmed
ACE10 <- Adverse Childhood Experiences	0.383	2.190	0.029	Confirmed
ACE11 <- Adverse Childhood Experiences	0.412	3.482	0.001	Confirmed
ACE2 <- Adverse Childhood Experiences	0.582	5.049	0.000	Confirmed
ACE3 <- Adverse Childhood Experiences	0.446	3.567	0.000	Confirmed
ACE4 <- Adverse Childhood Experiences	0.595	5.453	0.000	Confirmed
ACE5 <- Adverse Childhood Experiences	0.789	10.077	0.000	Confirmed
ACE6 <- Adverse Childhood Experiences	0.331	2.787	0.004	Confirmed
ACE7 <- Adverse Childhood Experiences	0.494	4.143	0.000	Confirmed
ACE8 <- Adverse Childhood Experiences	0.305	0.006	0.000	Confirmed
ACE9 <- Adverse Childhood Experiences	0.370	2.893	0.008	Confirmed
AP <- Schema Modes	0.839	21.063	0.000	Confirmed
AS <- Schema Modes	0.892	22.775	0.000	Confirmed
BIEN <- Schema Modes	0.778	19.046	0.000	Confirmed
BOZRGS <- Schema Modes	0.456	5.043	0.000	Confirmed
GZ <- Schema Modes	0.720	13.047	0.000	Confirmed
JA1 <- Parent-Child Relationship	0.554	2.524	0.013	Confirmed
JA10 <- Parent-Child Relationship	0.719	2.020	0.043	Confirmed
JA11 <- Parent-Child Relationship	0.660	2.956	0.005	Confirmed
JA12 <- Parent-Child Relationship	0.621	2.676	0.009	Confirmed
JA13 <- Parent-Child Relationship	0.818	2.376	0.018	Confirmed
JA14 <- Parent-Child Relationship	0.848	2.463	0.014	Confirmed
JA15 <- Parent-Child Relationship	0.754	2.157	0.031	Confirmed
JA16 <- Parent-Child Relationship	0.838	2.437	0.015	Confirmed
JA2 <- Parent-Child Relationship	0.552	2.390	0.165	Confirmed
JA3 <- Parent-Child Relationship	0.572	2.711	0.009	Confirmed
JA4 <- Parent-Child Relationship	0.621	2.854	0.006	Confirmed
JA5 <- Parent-Child Relationship	0.446	2.366	0.017	Confirmed
JA6 <- Parent-Child Relationship	0.577	2.551	0.012	Confirmed
JA7 <- Parent-Child Relationship	0.644	2.818	0.007	Confirmed
JA8 <- Parent-Child Relationship	0.720	2.076	0.038	Confirmed
JA9 <- Parent-Child Relationship	0.621	2.754	0.080	Confirmed
KHARAM <- Schema Modes	0.394	3.489	0.000	Confirmed
KHODBM <- Schema Modes	0.722	10.407	0.000	Confirmed
MBIT <- Schema Modes	0.649	11.804	0.000	Confirmed
SHAD <- Schema Modes	0.309	2.107	0.268	Confirmed
TAK <- Schema Modes	0.645	10.957	0.000	Confirmed
TSHM <- Schema Modes	0.510	6.571	0.000	Confirmed
VALDPORT <- Schema Modes	0.688	9.838	0.000	Confirmed
VALDTANBIH <- Schema Modes	0.783	13.096	0.000	Confirmed
ZORGO <- Schema Modes	0.755	13.064	0.000	Confirmed

AVE and CR Values: All AVE values surpassed the recommended threshold of 0.50 **(**ACE = 0.558, Schema Modes = 0.543, Parent–Child Relationship = 0.548), and CR values ranged between 0.737 and 0.927, exceeding the 0.70 criterion. Cronbach’s alpha coefficients also demonstrated satisfactory internal consistency for all constructs (as seen in Table 8).

**Table 8:** Reliability and convergent validity indicators of the measurement model. This table reports the crucial reliability metrics, including Cronbach's Alpha (alpha) and Composite Reliability (CR), along with the Average Variance Extracted (AVE) for each latent construct. Values of CR >0.70 and AVE >0.50 are used to confirm the internal consistency and convergent validity of the scales

Validity Indicators	Cronbach's Alpha	Composite Reliability	Average Variance Extracted (AVE)
Adverse Childhood Experiences	0.755	0.737	0.558
Schema Modes	0.862	0.884	0.543
Parent-Child Relationship	0.958	0.927	0.548

### Discriminant validity

Discriminant validity was assessed using the Fornell–Larcker criterion, ensuring conceptual distinctiveness among constructs. The square root of each construct’s AVE (diagonal values: ACE = 0.747, Schema Modes = 0.737, Parent–Child Relationship = 0.740**)** exceeded the inter-construct correlations (as seen in Table 9), confirming that the constructs are empirically distinct. Overall, the measurement model demonstrated robust psychometric properties, providing a sound basis for structural analysis.

**Table 9:** Divergent validity analysis and inter-construct correlation matrix. This table presents the inter-construct correlation matrix. Divergent validity is assessed using the Fornell-Larcker Criterion, where the square root of the AVE (shown on the diagonal) must be greater than the correlation coefficients with all other constructs in its respective row and column. All values satisfy the criterion, confirming the distinctiveness of the constructs

Adverse Childhood Experiences	Schema Modes	Parent-Child Relationship
Adverse Childhood Experiences	0.747	
Schema Modes	0.434	0.737
Parent-Child Relationship	0.164	0.090

### Structural model assessment

Following the validation of the measurement model, the structural model was evaluated for its explanatory power (R^2^), predictive relevance (Q^2^), and overall fit.

### Explanatory and predictive power

Coefficient of Determination (R^2^): The R^2^ indicates the percentage of variance in the dependent variables explained by the independent variables. achieved an Schema-related modes R^2^ of 0.181 (Correlation Coefficient 0.189)(as seen in Table 10), This value indicates that the independent and mediator variables account for 18.1% of the variance in Schema-related modes, which is considered a moderate effect in the behavioral sciences [29].

**Table 10:** Coefficient of determination (R2) for endogenous constructs. This table reports the R2 values for the endogenous (dependent) variables in the model, specifically Schema Modes and family emotional climate

Construct	Correlation Coefficient	Coefficient of Determination (R^2^)
Schema-related mindsets	0.189	0.181
Parent-child emotional relationship (mother-child & father-child)	0.027	0.022

The R^2^ value indicates the proportion of the variance in the construct that is explained by its predictor variables, serving as a measure of the model's explanatory power

### Q^2^ criterion (predictive relevance)

The Q^2^ criterion (Stone and Geisser, 1975) determines the model’s predictive power. Q^2^ Criterion (Predictive Relevance): The Q^2^ criterion (Stone and Geisser, 1975) determines the model’s predictive power. The model’s cross-validated redundancy (Q^2^) was assessed (as seen in Table 11). The Q^2^ value for Schema-related modes was 0.225. Given the established thresholds of 0.02 (weak), 0.15 (moderate), and 0.35 (strong) predictive power, this indicates a moderate predictive relevance [30].

**Table 11:** Predictive relevance (Q2) of the research model constructs. The table presents the Stone-Geisser's Q2 values, calculated using the blindfolding procedure

Construct	Q^2^ =1−SSE/SSO
Schema-related mindsets	0.225
Parent-child emotional relationship (mother-child & father-child)	0.436
Average	0.331

Q^2^ values greater than zero indicate that the model has predictive relevance for the respective endogenous constructs, demonstrating the model's ability to predict data points not used in the estimation

### Overall model fit (GOF Criterion)

Although there is no universal consensus on a single fit index for PLS models, the Goodness**-**of**-**Fit **(**GOF**)** statistic was calculated as the geometric mean of AVE and R². The obtained GOF value of 0.282 (as seen in Table 12) falls within the *moderate* range (≥ 0.25), suggesting acceptable overall model fit and adequate explanatory quality [31]. Once the structural relationships were established, hypothesis testing was performed to assess the significance and strength of each proposed path.

**Table 12:** Structural model results and hypothesis testing for the full sample (*N*=100) using PLS-SEM

Construct	R^2^	AVE​
Adverse childhood experiences	–	0.747
Schema-related mindsets	0.189	0.737
Parent-child emotional relationship (mother-child & father-child)	0.027	0.740
Average	0.108	0.741

Presents the results of the Partial Least Squares Structural Equation Modeling (PLS-SEM) analysis for the full sample, including *T*-Values, *P*-Values, and the outcome of the hypothesis test (Supported or Not Supported) for the hypothesized paths

### Hypothesis testing

**Fig. 4:**
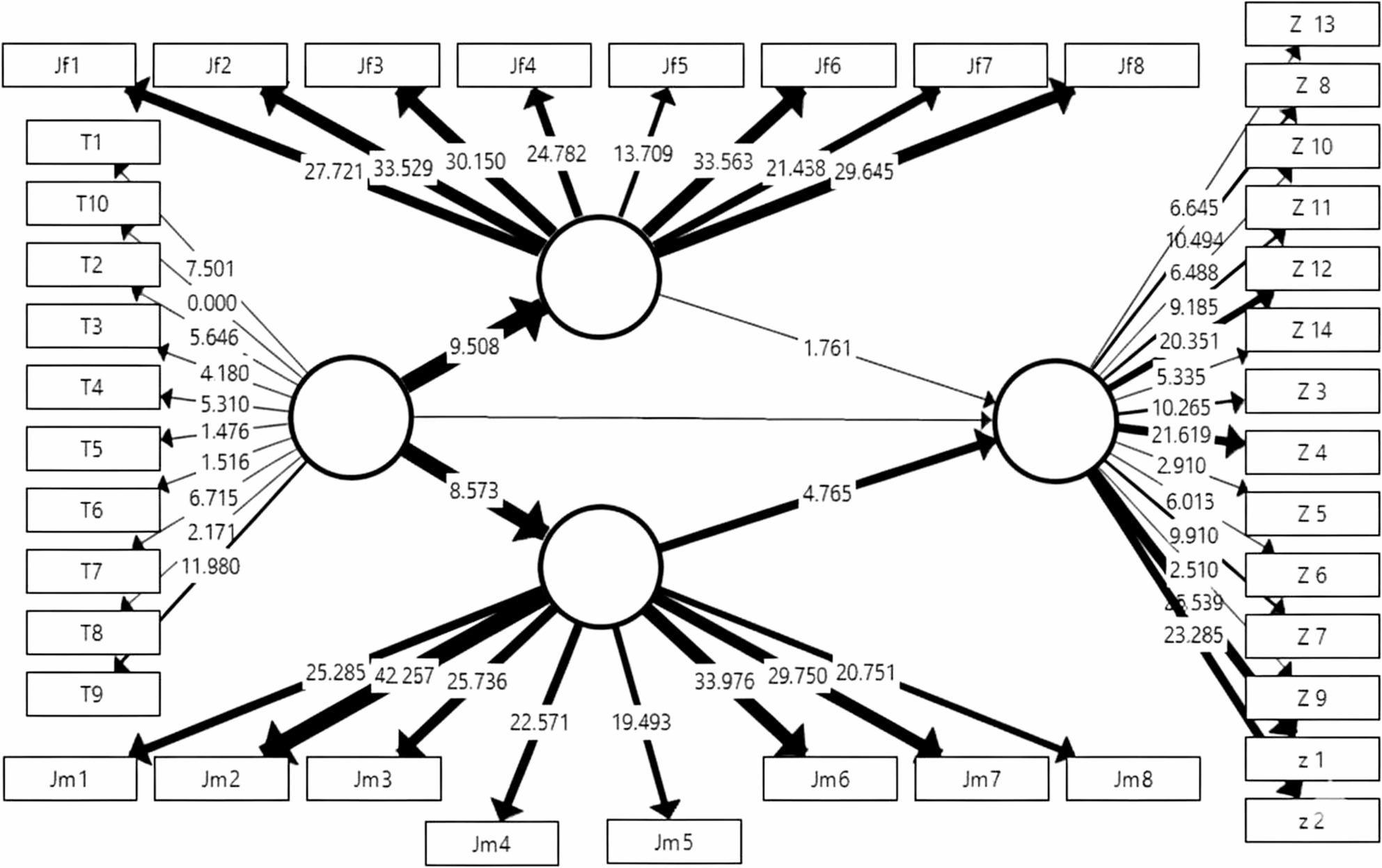
Final Structural Equation Model (PLS-SEM) presenting *T*-values for the total sample (*N*=100). The figure illustrates the hypothesized structural relationships among Adverse Childhood Experiences (ACEs), Family Emotional Climate (FEC), and Schema Modes. The corresponding *T*-Values, calculated using the 5,000 subsample bootstrapping procedure. Solid lines indicate statistically significant paths (*p* <.05) and dashed lines indicate non-significant paths. The model explains 18% of the variance in Schema Modes

**Fig. 5:**
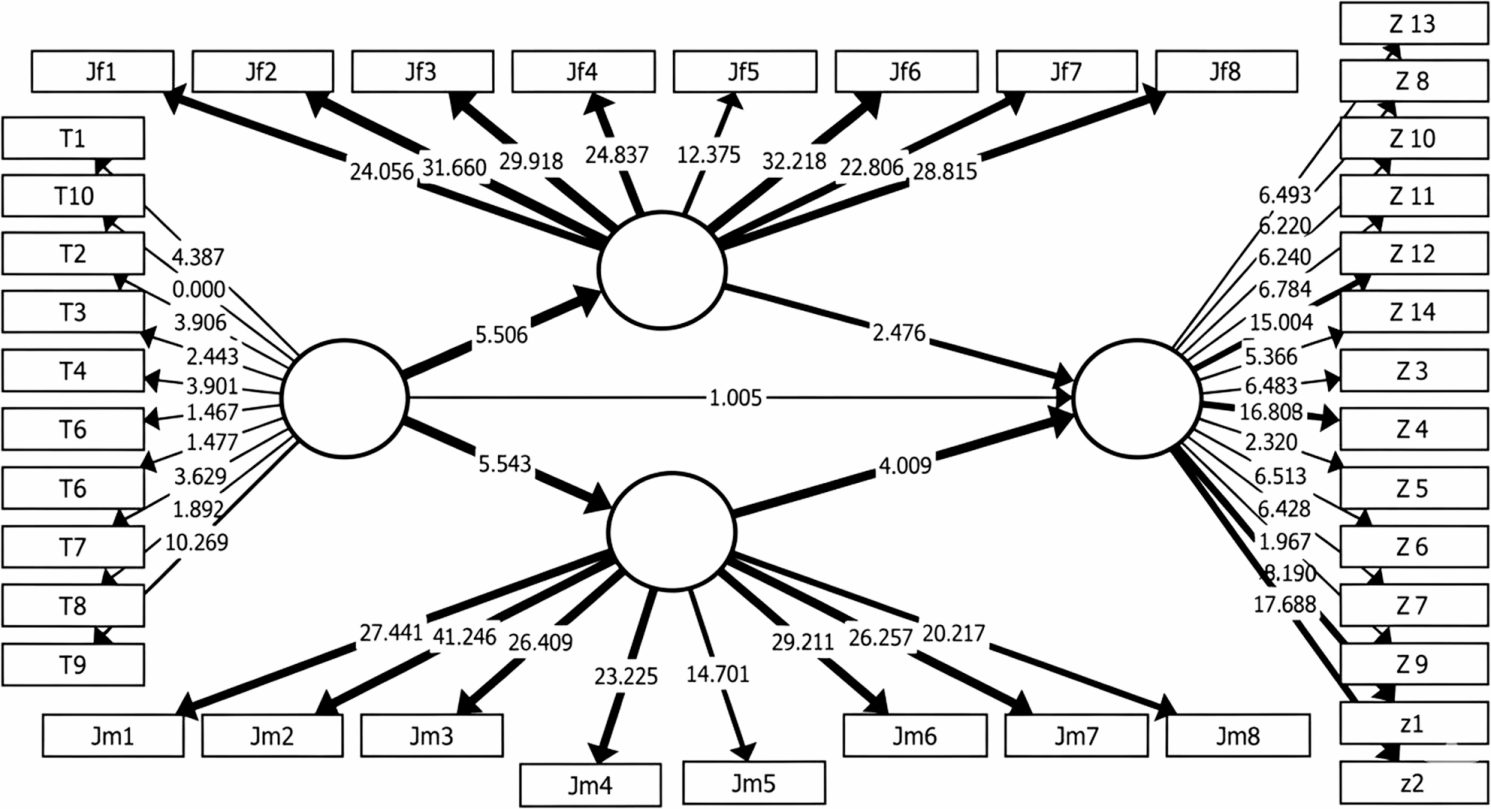
Final Structural Equation Model (PLS-SEM) presenting *T*-Values for the daughters subgroup (*N* = 77). The model illustrates the hypothesized relationships among Adverse Childhood Experiences (ACEs), Family Emotional Climate (FEC), and Schema Modes specifically for the female participants. The corresponding T-Values, calculated using the 5,000 subsample bootstrapping procedure. Paths that are statistically significant (*T* >1.96 or *p* <.05) are indicated by solid lines, highlighting the unique predictive roles of mother-child and father-child relationships in this subgroup

**Fig. 6:**
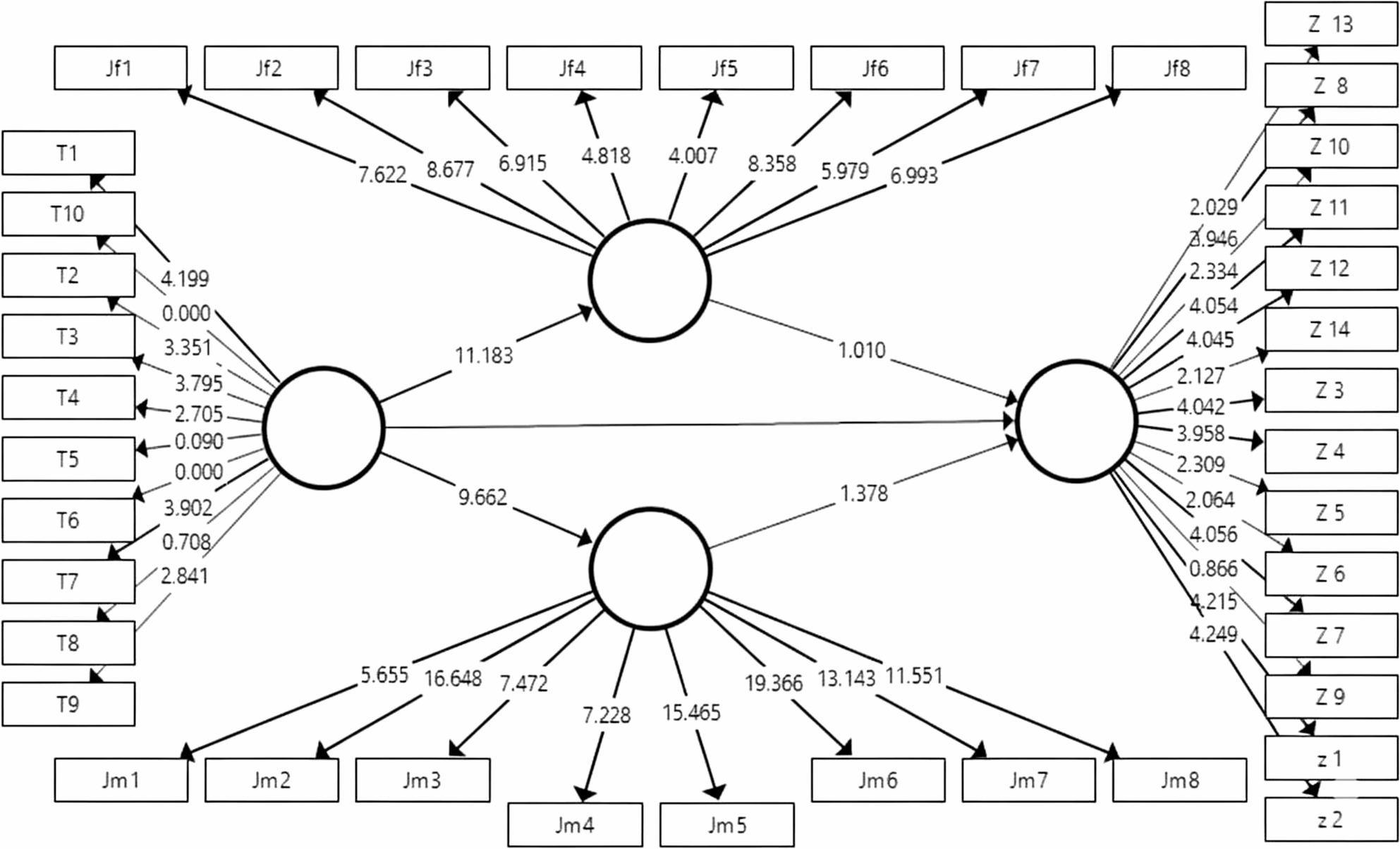
Final Structural Equation Model (PLS-SEM) *T*-Values for the sons subgroup (*N* =23). The model illustrates the hypothesized structural relationships among Adverse Childhood Experiences (ACEs), Family Emotional Climate (FEC), and Schema Modes specifically for the male participants. The corresponding *T*-Values, calculated using the 5,000 subsample bootstrapping procedure. Paths that are highlighting the unique predictive roles of mother-child and father-child relationships in this subgroup. No significant paths emerged in this subgroup (all *p* >.05)

Hypothesis testing was performed using a non-parametric bootstrapping procedure with 5,000 resamples, to ensure robust estimation of standard errors and significance levels, following recommended guidelines of Hair et al. study [29]. The structural equation model and path diagrams, including significance values and path coefficients, are illustrated in Figs. 4, 5 and 6.

### Overall sample results

The examination of the proposed relationships for the overall sample revealed the following:

The paths from Adverse childhood experiences to both the Mother-child emotional relationship (t = 8.57, *p* <.001) and the Father-child emotional relationship (t = 9.50, *p* <.001) were strongly supported. The direct path from Adverse childhood experiences to Schema-related modes was not supported (t = 1.124, non-significant). The mediating path from Mother-child emotional relationship to Schema-related modes was significantly supported (t = 4.76, *p* <.001). The mediating path from Father-child emotional relationship to Schema-related modes was not supported (non-significant) (as seen in Table 13; Fig. 4). Beyond the overall model evaluation, additional analyses were conducted to explore potential gender-based variations in the structural relationships.

**Table 13:** Structural model results and hypothesis testing for total sampel using PLS-SEM

Path	β (Path Coefficient)	SE (Standard Error)	*t*-Value	*p*-Value	Result
ACEs → Mother-Child Relationship	−0.566	0.066	8.573	<.001	Supported
ACEs → Father-Child Relationship	−0.67	0.089	7.492	<.001	Supported
ACEs → Schema Mentality	0.135	0.084	1.601	0.109	Not Supported
Mother-Child Relationship → Schema Mentality	−0.407	0.102	4	<.001	Supported
Father-Child Relationship → Schema Mentality	−0.274	0.111	2.472	0.013	Supported

PLS-SEM was performed using 5,000 bootstrap resamples. β indicates the standardized path coefficient. SE is the standard error. *T* -Values greater than 1.96 are considered statistically significant at *p* < .05

### Subgroup analysis (Gender)

The relationships were examined separately for male and female participants (as seen in Figs. 5 and 6; Tables 14 and 15) revealed the following: For daughters (Female Participants), The results indicated strong statistical significance for all relationships except the direct path from Childhood Adverse Experiences to Schema Modes (t = 1.60; Not Confirmed). Specifically, the path from Emotional Climate (Father-Child Relationship to Schema Modes was confirmed (t = 2.47, *p* <.001), and for Sons (Male Participants), none of the structural paths from ACEs or from parental emotional climate constructs to schema modes reached statistical significance (all t-values < 1.96, *p* >.05). Although ACEs were associated with mother–child and father–child emotional climate scores, these associations did not translate into significant relationships with schema modes within the male subgroup. Therefore, the present data do not support definitive conclusions about the impact of ACEs or parental emotional relationships on schema modes among male participants (t < 1.96, Not significant) (as seen in Figs. 5 and 6; Tables 14 and 15).

**Table 14:** Structural Model Results and Hypothesis Testing for the daughter subgroup using PLS-SEM

Path	β (Path Coefficient)	SE (Standard Error)	*t*-Value	*p*-Value	Result
ACEs → Mother-Child Relationship	−0.534	0.096	5.543	<.001	Supported
ACEs → Father-Child Relationship	−0.593	0.078	7.601	<.001	Supported
ACEs → Schema Mentality	0.063	0.103	0.613	0.54	Not Supported
Mother-Child Relationship → Schema Mentality	−0.467	0.137	3.411	<.001	Supported
Father-Child Relationship → Schema Mentality	−0.246	0.138	1.783	0.075	Not Supported

PLS-SEM was performed using 5,000 bootstrap resamples for the daughters subgroup.β indicates the standardized path coefficient. SE is the standard error. *T* -Values greater than 1.96 are considered statistically significant at *p* < .05

**Table 15:** Structural model results and hypothesis testing for the sons subgroup

Path	β (Path Coefficient)	SE (Standard Error)	*t*-Value	*p*-Value	Result
ACEs → Mother-Child Relationship	−0.733	0.076	9.662	<.001	Supported
ACEs → Father-Child Relationship	−0.796	0.071	11.185	<.001	Supported
ACEs → Schema Mentality	0.128	0.096	1.34	0.18	Not Supported
Mother-Child Relationship → Schema Mentality	−0.134	0.098	1.365	0.172	Not Supported
Father-Child Relationship → Schema Mentality	−0.1	0.099	1.006	0.314	Not Supported

PLS-SEM was performed using 5,000 bootstrap resamples for the sons subgroup.β indicates the standardized path coefficient. SE is the standard error. *T* -Values greater than 1.96 are considered statistically significant at *p* < .05

These findings validate the proposed structural model and emphasize the utility of the PLS approach in interpreting the phenomenon under study, highlighting distinct gender-based differences in the mediation process. Overall, the findings provide empirical support for the proposed model and suggest that early adverse experiences influence cognitive schemas indirectly through the quality of parent–child emotional relationships. These statistical findings provide the foundation for a deeper theoretical interpretation, which is discussed in the following section.

## Discussion

Parental behaviors, particularly in relation to gender, substantially shape cognitive and emotional development across the lifespan [32–35]. Prior research suggests that connections between mothers and children matter more when problematic thinking patterns develop than those with fathers. While Young’s original ideas about these thought patterns are still important, newer studies explore them alongside today’s world and different cultures [5]. Arntz and van Genderen (2020) pointed out schema modes aren’t just fleeting feelings or thoughts; they are evolving relationships originating in childhood. Consequently, this idea supports our investigation into how a parent’s gender shapes these schemas [36].

Studies now show how bonds with parents, alongside their approaches to raising children, contribute to how males versus females develop certain thought patterns. For instance, Louis and colleagues (2022) discovered that showing affection plus offering support impacts boys and girls differently—mothers and fathers seem to satisfy unique emotional requirements [37]. Likewise, Pilkington et al.’s (2021) broad look at existing research revealed variations in mothering and fathering strongly correlate with the development of unhelpful beliefs formed early on, though how a parent’s own gender plays into this isn’t fully understood—something our work seeks to clarify [38].

Adverse early-life experiences, particularly emotional neglect or lack of affection, are associated with the development of maladaptive cognitive patterns because they damage connections with parents. It’s becoming clear we need both established ideas about these patterns alongside newer understandings from child development and diverse cultures [39, 40]. This research adds to that conversation by indicating that differences in mother–child versus father–child relationships—shaped by traditional parenting expectations—may help contextualize the lasting effects of early adversity. Jeffrey Young’s initial work on Schema Therapy revealed how troublesome patterns—rooted in childhood—develop from difficult beginnings. Specifically, these patterns stem from what happens when a children’s natural personality clashes with hurtful experiences, notably feeling uncared for by parents. Meanwhile, Bernstein alongside Fink offered further evidence linking painful events to the way we think [3, 11, 41]. Difficult early life events seem linked to how people develop thinking patterns—a connection backed by statistical evidence (*p* <.05). These results align with ideas from Schema Therapy, alongside previous work on the subject [8, 9].What was specifically explored in this study was the types of schema modes that dominate individuals’ minds, which had not been previously examined in detail. Although earlier studies examined the relationship between negative childhood experiences and specific schemas, such as emotional neglect with emotional deprivation and social isolation schemas, and emotional abuse with mistrust/abuse and defect/shame schemas, this study identified a broader spectrum of schema modes [42–48]. The main schema modes identified as most dominant in this study, based on regression-derived t-values, were the Vulnerable Child, Angry Child, Impulsive Child, and Punitive Parent (Table 5). Table 5 presents both these t-values and the corresponding raw mean scores for each mode, allowing a comparison between model-based prominence and descriptive scores. Although these modes were identified as dominant according to t-values, their ranking based on mean scores differed. This divergence is expected, as mean scores reflect the overall intensity of each mode in the sample, whereas t-values indicate the unique contribution of each mode when controlling for other modes. Presenting both indices provides complementary insights into the relative prominence of schema modes and ensures transparency in the interpretation of the findings. The main schema modes with the highest scores in this study were the vulnerable child, the angry child, the impulsive child, and the punitive parent. The vulnerable child mode reflects feelings of helplessness, fear, and incapacity that a child experiences when faced with unhealthy and unstable environments. In such situations, children feel unprotected and unsupported. This schema typically forms after repeated failures to receive emotional support from parents. In adulthood, individuals with this schema may be highly sensitive and vulnerable to challenges and struggle to cope with life’s difficulties.

In contrast, the Angry child mode represents suppressed emotions of anger and frustration. Children raised in environments with high control or violence often suppress their angry feelings, which later manifest as maladaptive behaviors. This schema may lead to aggressive behaviors or extreme reactions to stressors in adulthood.

The impulsive child mode, meanwhile, is linked to unplanned behaviors and unpredictable, impulsive reactions. Children raised in unstable environments or without adequate self-control training may struggle to regulate their behavior in adulthood. These individuals might react impulsively to strong emotions and fail to consider the consequences of their actions.

Finally, the punitive parent mode reflects the internalization of harsh, punitive behaviors that children observe in their interactions with parents. Children raised by punitive parents internalize these patterns and may unconsciously replicate them in their interactions with others, particularly their own children, in adulthood. These individuals often display behaviors that perpetuate cycles of violence and blame.

One of the noteworthy findings of this study was the difference in the impact of the mother-child and father-child relationships on cognitive schemas. While the emotional tone of the mother-child relationship had a significant association with cognitive schemas (*p* <.05), this relationship was not significant in the case of father-child interactions (*p* >.05). This finding warrants exploration from several perspectives:

In many cultures, mothers play the primary role in nurturing and caregiving, and this close and continuous relationship has a stronger impact on the formation of a child’s emotional and cognitive patterns [48, 49]. Mothers tend to express emotions more openly than fathers, which may influence how children learn to express and manage emotions. Attachment theorists believe that attachment quality with mothers plays a more significant role in a child’s emotional and cognitive development. This may explain why mother-child relationships have a stronger influence on cognitive schemas [5].

In some cultures, fathers’ roles may be more associated with financial support, while mothers are seen as the primary caregivers. These cultural patterns could influence the outcomes of this study. The lack of significance in father-child relationships may reflect cultural, social, and traditional parental roles in the studied community. A study by Cassidy and Shaver found that mothers play a key role in creating secure attachment and providing emotional support, while fathers often fulfill social and cultural model roles [12].

Although cultural literature highlights the importance of paternal authority and social modeling, the present findings did not show a significant association between father–child emotional relationship scores and schema modes. As a result, interpretations about paternal influence on schema mode development should remain cautious and cannot be inferred directly from the statistical pathways tested in this model.

Because no significant paths to schema modes emerged for male participants, any assumptions regarding stronger paternal influence on boys—such as identification needs or father-specific emotional dependence—should be considered exploratory rather than empirically supported by the current data.

Likewise, statements about specific emotional needs that boys may seek from fathers (e.g., nurturing, encouragement, or approval) cannot be concluded from the present model, as the father–child emotional relationship did not demonstrate significant predictive value for schema modes.

### Emotional and psychological needs by gender

Although descriptive tendencies may exist in father–child emotional patterns across genders, these should not be interpreted as confirmed differences in the development of schema modes. The non-significant pathways for male participants prevent drawing firm conclusions about gender-specific emotional needs related to paternal relationships.

### Gender differences in cognitive schemas

Although some descriptive differences in schema mode scores were observed between boys and girls, the absence of significant structural paths in the male subgroup means that interpretations regarding gender-specific schema dominance should remain cautious and descriptive rather than inferential.

Overall, the results suggest that boys and girls react differently to negative childhood experiences. These differences can be attributed to cultural gender roles, social expectations, and parental behavioral patterns. Boys, traditionally seen as symbols of power and resilience, may develop passive behaviors as a defense against emotional challenges, while girls, who are more socially expected to express emotions, may exhibit impulsive behaviors in response to stressors.

### Differential impact of parental gender 

One of the notable findings of this study was the differential impact of mother-child versus father-child relationships on the development of schema modes. Specifically, the emotional quality of mother-child interactions showed a significant association with schema modes (*p* <.05), whereas father-child interactions did not reach statistical significance (*p* >.05). This pattern may be interpreted from multiple, interrelated perspectives.

First, maternal caregiving often constitutes the primary source of emotional support in many cultures, providing continuous and attuned responses that strongly shape children’s cognitive and emotional patterns. Second, attachment theory emphasizes the central role of early maternal bonds in regulating emotions and fostering adaptive cognitive schemas, which may explain the stronger influence of mother-child relationships on schema modes. Finally, cultural and social norms may contribute to these findings, as fathers’ roles in the studied community are often more closely linked to financial provision than to daily emotional caregiving. Taken together, these results suggest that the formation of early maladaptive schemas is not only biologically and psychologically grounded but also embedded within the sociocultural context of parenting, highlighting the need for schema-focused interventions to consider both relational and cultural dimensions.

## Conclusion

Taken together, these findings highlight both theoretical and practical implications, summarized as follows: childhood cognitive schemas can have profound effects on individuals’ personalities and behaviors in adulthood. These schemas act as persistent patterns in cognition, emotions, and behaviors, and can negatively impact personal and social relationships. Individuals who experience negative childhood events and form maladaptive schemas may struggle with intimate and healthy relationships and face emotional and social difficulties due to insecurity, anger, and mistrust.

The results suggest that parental interaction with children, particularly the emotional quality of the mother–child relationship, influences psychological growth and the formation of cognitive schemas. This finding underscores the significant role of the maternal emotional bond, while the non-significant findings regarding the father–child relationship and the lack of significant paths in the male subgroup suggest caution when interpreting the broader influence of parental gender on schema development. Maladaptive schemas, such as the vulnerable child and the angry child, arise from unmet psychological needs and can have detrimental effects on an individual’s mental and behavioral health throughout their life. The observed differences in the predictive power of maternal versus paternal relationships highlight the necessity for parents to be mindful of the primary role of maternal emotional support in the context of schema formation. While the findings offer meaningful insights, certain methodological and contextual limitations should be acknowledged.

## Limitations and future research

### Limitations

We acknowledge that the present study possesses certain limitations, The principal limitation of this study lies in its cross-sectional design, which captures participants’ responses at a single point in time. While such a design is valuable for identifying associations among variables, it inherently constrains the ability to determine temporal precedence and to infer causality. Consequently, it remains unclear whether adverse childhood experiences precede and shape specific patterns of parental relationships and schema modes, or whether these relationships evolve through reciprocal and dynamic interactions over time. Moreover, the absence of longitudinal data limits our understanding of developmental trajectories and potential mediating processes. Future research employing longitudinal or experimental designs would enable a more precise examination of these causal pathways and contribute to a deeper understanding of the mechanisms linking early adverse experiences to schema development.

The second limitation of our study is its regional focus on Sanandaj, which may constrain the broader applicability of our findings. Cultural norms play a crucial role in shaping parenting behaviors and emotional expression, and these factors can vary considerably across regions. For instance, Li et al. (2025) reported notable differences in how parents in France and the United States respond to children’s negative emotions, demonstrating that socioemotional outcomes are deeply influenced by culturally specific parenting practices. Taken together, these insights underscore the need for caution when generalizing our results beyond the sampled region, and highlight the importance of considering cultural context in interpreting the observed relationships [50].

The third limitation of our study pertains to the exclusive reliance on self-report questionnaires for data collection. While self-reports are a widely used and efficient method, they are susceptible to several biases that can compromise the validity of the findings. Specifically, common method bias may inflate associations between predictor and outcome variables when both are assessed using the same method. Additionally, recall bias can affect the accuracy of participants’ retrospective accounts, particularly concerning complex or latent aspects of childhood experiences. Recent research underscores these concerns: Trott (2025) emphasizes that retrospective self-reports in studies of childhood maltreatment are prone to common method bias, potentially distorting results [51] Furthermore, LaNoue (2024) discusses how both epidemiologic and psychometric approaches recognize the limitations of self-reported data, highlighting the importance of cautious interpretation [52].

The fourth limitation is that Although we conducted separate subgroup analyses by gender, this study did not formally test the statistical significance of path coefficient differences between subgroups using PLS-MGA. Future studies should employ formal multi-group analysis to confirm these observed gender differences.

Finally, this study focused on individuals currently receiving psychotherapy, which may limit how widely the findings apply. These participants might have higher levels of schema activation or distress. Moreover, factors like mental health status, socioeconomic background, and therapy duration were not controlled, which could influence results. Future studies should include more diverse samples and account for these variables.

### Future research

Based on these limitations, future research should consider the following recommendations, first Employing longitudinal designs is strongly recommended to better understand the developmental trajectories and causal relationships between adverse childhood experiences and schema mode formation over time. Longitudinal approaches allow researchers to track changes in schema modes and related psychological outcomes, providing stronger evidence of causality [53], Second, Incorporating multi-method strategies, such as observational assessments of parent-child interactions and structured clinical interviews, would offer a more comprehensive evaluation of childhood experiences. Such approaches reduce reliance on a single data source and mitigate potential common method bias [54], Third one is Expanding research to include more culturally and geographically diverse samples is essential to enhance the generalizability of findings and to explore how cultural contexts may influence parenting practices and schema development. Cross-cultural studies have demonstrated that both childhood experiences and schema formation can vary significantly across cultural settings [55].

## Data Availability

The datasets used and analyzed during the current study are available from the corresponding author on reasonable request.

## Abbreviations

DSM: The Diagnostic and Statistical Manual of Mental Disorders
ACEs: Adverse Childhood Experiences
PLS: Partial Least Squares
SEM: Structural equation modeling
(K-S): Kolmogorov-Smirnov test
Sig: Significance level
AVE: Average Variance Extracted
CR: Composite reliability
GOF: Goodness-of-fit index
